# Online measurement of learning temporal statistical structure in categorization tasks

**DOI:** 10.3758/s13421-022-01302-5

**Published:** 2022-04-04

**Authors:** Szabolcs Sáringer, Ágnes Fehér, Gyula Sáry, Péter Kaposvári

**Affiliations:** Department of Physiology, Albert Szent-Györgyi Medical School, Faculty of Medicine, University of Szeged, 10. Dóm tér, Szeged, 6720 Hungary

**Keywords:** Visual statistical learning, Motor learning, Temporal dynamics, Anticipation, Priming

## Abstract

The ability to grasp relevant patterns from a continuous stream of environmental information is called statistical learning. Although the representations that emerge during visual statistical learning (VSL) are well characterized, little is known about how they are formed. We developed a sensitive behavioral design to characterize the VSL trajectory during ongoing task performance. In sequential categorization tasks, we assessed two previously identified VSL markers: priming of the second predictable image in a pair manifested by a reduced reaction time (RT) and greater accuracy, and the anticipatory effect on the first image revealed by a longer RT. First, in Experiment 1A, we used an adapted paradigm and replicated these VSL markers; however, they appeared to be confounded by motor learning. Next, in Experiment 1B, we confirmed the confounding influence of motor learning. To assess VSL without motor learning, in Experiment 2 we (1) simplified the categorization task, (2) raised the number of subjects and image repetitions, and (3) increased the number of single unpaired images. Using linear mixed-effect modeling and estimated marginal means of linear trends, we found that the RT curves differed significantly between predictable paired and control single images. Further, the VSL curve fitted a logarithmic model, suggesting a rapid learning process. These results suggest that our paradigm in Experiment 2 seems to be a viable online tool to monitor the behavioral correlates of unsupervised implicit VSL.

**Supplementary Information:**

The online version contains supplementary material available at 10.3758/s13421-022-01302-5.

## Introduction

During perception and sensory learning, we can extract contextually relevant information from the environment. Due to the infovore nature of our sensory system, it is capable of dividing the continuous information stream into chunks and grasping relations between them (Biederman & Vessel, 2006). The implicit process by which these patterns are recognized and built into internal representations is called statistical learning (SL).

This approach was used initially in studies related to language acquisition (Saffran, Aslin, & Newport, 1996a; Saffran, Newport, & Aslin, 1996b), where segmentation of sensory streams is described as “the use of regularities to parse the world into meaningful lexical units” (Giroux & Rey, 2009). Additionally, SL has been demonstrated in audition (Saffran et al., 1999) and haptics (Conway & Christiansen, 2005), and studied extensively regarding vision in the context of spatial (Fiser & Aslin, 2001; Fiser & Aslin, 2005) and temporal regularities (Fiser & Aslin, 2002; Kaposvari et al., 2018; Meyer et al., 2014; Olson & Chun, 2001), including in multisensory contexts (Seitz et al., 2007).

Initial studies employed a passive familiarization phase during which a continuous stream of stimuli was presented to participants, followed by an “offline” post-presentation test of learning outcome (Fiser & Aslin, 2001; Fiser & Aslin, 2002; Fiser & Aslin, 2005; Saffran et al., 1999). During the familiarization phase, participants either pay attention passively or perform unrelated cover tasks so that the learning process is supposed to be implicit. The most frequently used offline measurement of SL is the familiarity test where the participants have to choose between the earlier presented patterns and randomly generated (novel) patterns. This paradigm provides information about the learning outcome, but not about the progression of learning, the strategies used, or the attention of subjects (Barakat et al., 2013; Bertels et al., 2012; Jonaitis & Saffran, 2009.

The world around us is changing dynamically, therefore it is necessary to make continuous inferences and adapt our knowledge accordingly. Thus, perception consists of not a series of static snapshots of the surroundings, but rather an ever-changing environmental model that is constantly updated based on the successes and errors of its predictions (Maloney & Mamassian, 2009). SL studies have provided evidence that the measured SL is the result of a complex dynamic process involving perceptual learning (Barakat et al., 2013; Fiser & Lengyel, 2019). However, the dynamics of learning cannot be investigated with those initial approaches using “static” offline measurements focusing only on the learning outcome. Thus, there is a compelling need for online measurement of SL to investigate this complex learning process. Such an online approach could also provide information about how a statistical pattern is gradually integrated into the representation, and how predictions influence perception. Additionally, online measurements with unrelated parallel tasks may be superior for controlling the level of attention, which is essential for SL (Turk-Browne et al., 2005), compared to offline studies using a passive fixation paradigm during which the level of attention may vary widely. First, parallel tasks occupy participants and help to keep the task implicit. Second, since the task is object related, it focuses attention on the stimuli.

Previous studies of SL have focused on language acquisition, and they represent use of explicit knowledge (Amato & MacDonald, 2010; Gómez et al., 2011; Misyak et al., 2010) as the participants knew that the syllables could be part of an artificial language and so actively searched for artificial words. This may result in more robust inferences compared to an implicit task design and even rely on distinct underlying mechanisms (Batterink, Reber, Neville, & Paller, 2015a; Batterink, Reber, & Paller, 2015b). Moreover, the higher level of attention of participants in an explicit procedure affects the resulting learning trajectory (Turk-Browne et al., 2005).

A few previous studies have examined visual statistical learning (VSL) online. For instance, Siegelman and colleagues used a self-paced paradigm (Karuza et al., 2014) to monitor the trajectory of VSL (Siegelman et al., 2018) and were able to describe the learning curve. However, the participants were informed before the test that the presented information stream contains regularities that may have affected the learning mechanism. Since explicit and implicit processes likely differ mechanistically, there is still a need for an online measurement task that assesses implicit SL. Turk-Browne et al. (2010) came closest to achieving online measurement of implicit SL. They measured VSL using a two-alternative forced-choice (2AFC) categorization task with associated stimulus pairs as the regularity during event-related functional magnetic resonance imaging (fMRI). As they applied a control condition consisting of single stimuli (i.e., stimuli with no statistical relation to any other stimuli), it was possible to identify two markers of learning: an effect of anticipation on the first stimulus of the pair as shown by a prolonged reaction time (RT), and a priming effect on the second stimulus of the pair relative to single stimuli manifested by a reduced RT.

Processing of the first image in a pair is affected because it has an anticipatory role through portending the next image in the stimulus pair without explicit knowledge of the subject. This effect has been characterized by a longer RT for this condition in that study. Since the second image is always preceded by the first, the second one becomes predictable, which can be monitored with a shortened RT. Familiarity testing was used to confirm learning at the end of each session (Turk-Browne et al., 2010).

However, the task used by Turk-Browne et al. (2010) may have been confounded by motor priming. In their paradigm, there were two categories (scenes and faces) and three conditions (first image of a pair, second image of a pair, and single image). In the stimulus pairs, the picture categories were always different (face-scene or scene-face). Therefore, the appropriate response was somewhat predictable since if the current picture is a face, there is about a 65% chance that the next one will be a scene, and this predictor becomes 100% accurate in the case of stimulus pairs. Also, repetition of a category can only occur in the case of a single image or the first image of a pair, prolonging their RTs. This unbalanced category transition could introduce additional procedural or motor learning.

Our main goal was to develop an online measurement to test the behavioral correlates of unsupervised implicit VSL and to characterize the learning process. We adapted the paradigm of Turk-Browne et al. (2010), and our first aim was to investigate the presence of a possible motor learning confound. After we confirmed and eliminated this confound by balancing the category transitions, the design showed a drastically reduced effect on anticipation and priming. Therefore, our next aim was to modify the paradigm in order to acquire sufficient data for measuring the priming and/or anticipation effects and tracking the learning process online. Using our novel design, we successfully demonstrated the priming effect. Moreover, our results suggest very fast learning dynamics. Overall, our paradigm could be a useful online measure of unsupervised, implicit VSL.

## Experiment 1A

Considering recent literature, we decided to reproduce the behavioral part of the experiment designed by Turk-Browne et al. (2010) with some modification, both to reveal the learning effect and to acquire sufficient data for characterization of the learning trajectory.

First, we increased the sample size (both the number of subjects and the number of runs) to increase the statistical power. Second, since the accuracy (ratio of correct answers) in the original study was not sensitive enough to show the anticipation or priming effect, we made the task more challenging by introducing a complex discrimination requirement for the different sets of stimuli and by increasing the stimulus duration while retaining the original regularity. These modifications reduced the ceiling effect and increased the sensitivity of categorization speed (RT) and accuracy as indicators of SL.

### Material and methods

#### Participants

Thirty-eight healthy right-handed volunteers with normal or corrected-to-normal vision participated in Experiment 1A (20 females, mean age: 25.34 years; range: 21–41 years). All provided written informed consent and the study protocol was approved by the Human Investigation Review Board of University of Szeged (266/2017-SZTE)

#### Stimuli

Complex gray-scale images of everyday objects were previously selected from the Bank of Standardized Stimuli (BOSS) (Brodeur et al., 2010; Brodeur et al., 2014). Selection was based on the results of a previous 2AFC discrimination task in which five subjects who were not participants of the current experiments were asked to decide whether a presented object fits into an imaginary shoebox (33 cm × 19 cm × 12 cm). Those stimuli for which the responses were concordant across the five participants were selected and labeled “Large” or “Small.” In Experiment 1A, 48 stimuli were used from both categories (96 distinct stimuli in total), of which 12 (six Large, six Small) were used for each of the eight runs constituting one experimental session. All stimuli subtended approximately 7.5° × 7.5° in visual angle.

#### Design

A sequence of these object images was presented to the participants, and behavioral responses (RT and accuracy) were recorded across eight runs. For one run, six-six randomly chosen images were used from both categories of the stimulus set to create a temporal sequence in a pseudo-random order. The same stimuli were never presented again in subsequent runs. The 12 stimuli formed information chunks consisting of four single stimuli and four associated stimulus pairs. One run contained a sequence of 72 trials divided into six cycles. In each cycle, all information chunks appeared once in random order with the constraint that at the juncture of cycles, a stimulus could be repeated only after three different stimuli had been presented. Thus, the stimulus pairs were well distributed within the sequences to control repetition effects.

#### Task and procedure

**Fig. 1:**
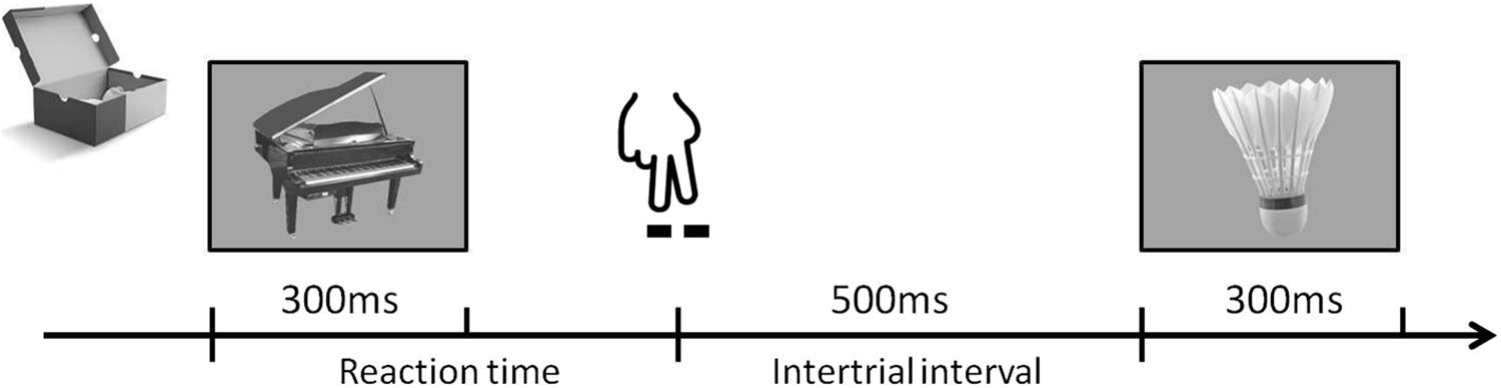
Schematic of the task trial design for Experiments 1A and 1B

The Serial Reaction Time (SRT) protocol was adapted from Turk-Browne et al. (2010) with modifications. The participants performed the discrimination task in a sound-attenuated room with dimmed light. They were requested to indicate whether the presented object could fit into a shoebox (was “Small”) by pressing button 1 with their right index finger or not (was “Large”) by pressing button 2 with their right middle finger on a numeric keyboard (Fig. 1). No feedback was provided during the task. The response accuracy (correct responses/total responses) and RTs were recorded. Stimuli were presented for 300 ms, and the next stimulus was presented only after the participant responded to the current stimulus (so trials were subject paced). The intertrial interval (ITI) was fixed to 500 ms. The experiment was run in Matlab (Mathworks, Natick, MA, USA) Psychtoolbox (Brainard, 1997) using an HP 650 ProBook G4 (screen: 15.6 in., resolution 1,920 × 1,080 pixels, 60 fps). Participants were seated approximately 60 cm from the screen. They were naïve to the pattern of the stimulus stream (see below for details of the pattern). After each experimental session, participants were asked the following questions to determine if the regularity was explicitly recognized: (1) What is your impression of the experiment? (2) Do you have any observations about the experiment? (3) Did you recognize any pattern regarding pressing the response keys? (4) Did you find any pattern regarding the images? (5) Did you find any systematic regularities about the order of the images?

RT and accuracy were measured in a discrimination task in which the participants were asked to categorize the presented objects according to real-world size as fitting in a shoebox (“Small,” as the badminton shuttlecock at right) or not (“Large” as the piano at left). The stimulus duration was 300 ms and a subsequent image was presented only after a response (subject paced). The intertrial interval was constant (500 ms).

#### Pattern

**Fig. 2:**
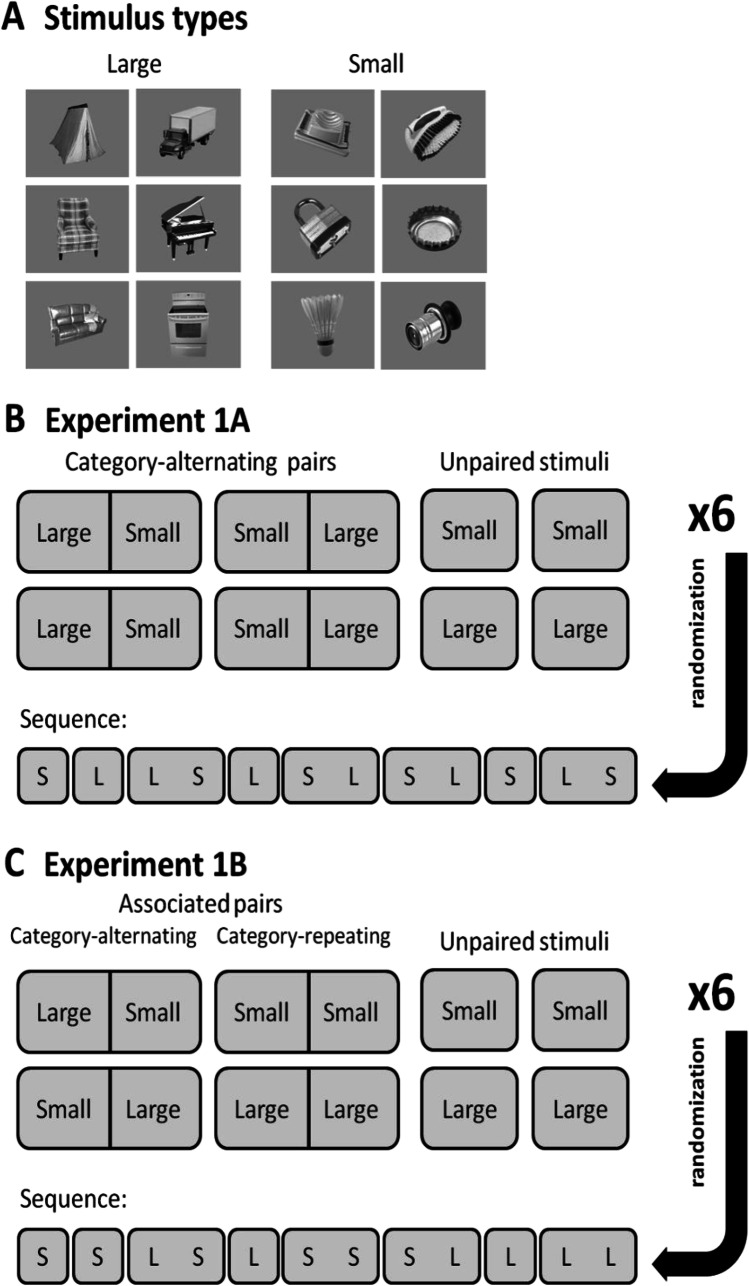
Schematics of the task designs for Experiment 1A and 1B. (**A**) Examples of randomly chosen stimuli from the Large and Small categories. (**B**) In Experiment 1A, four category-alternating stimulus pairs (Large–Small [L S] and Small–Large [S L]) were created, while the remaining four stimuli were unpaired. The constructed information chunks were used to create a 72-trial long sequence in each run. (**C**) In Experiment 1B, four associated pairs were created, two category-alternating pairs (Large–Small [L S] and Small–Large [S L]) and two category-repeating pairs (Large–Large [L L] and Small–Small [S S]). Four stimuli remained unpaired, two from the Large (L) category and two from the Small (S) category

To counterbalance the possible effects of individual stimuli, six stimuli were randomly chosen from both categories for each participant (Fig. 2A). Eight selected stimuli were grouped into associated pairs in fixed order. Here, we would like to introduce two definitions: category-repeating pair and category-alternating pair. By category-repeating pair, we mean that the categories of the images in the stimulus pair are the same (both do fit or do not fit into the shoebox), while category-alternating pair means the categories of the images are different in the pair (one of them fits into the box, the other does not). In Experiment 1A, pairs were category-alternating, while in Experiments 1B and 2, some were category-repeating. In Experiment 1A, two of the four pairs per run started with Small objects (Small–Large) and the other two with Large objects (Large–Small). The remaining two Large and two Small objects were presented as unpaired single stimuli. The stimulus combinations in the associated pairs formed the information chunks in the sequence (Fig. 2B).

#### Data processing

We measured RT and categorization accuracy as indices of performance. Accuracy was determined by concordance with the labels assigned during the aforementioned 2AFC experiment, where five participants’ concordant answers determined the label of the stimuli. Only data from correct answers were used to calculate average RTs. The following criteria were used to filter the trials for both the RT and accuracy analyses: responses slower than mean + 3 standard deviations (SDs) (following the exclusion criteria of the original study (Turk-Browne et al., 2010)) or faster than 200 ms were trimmed. All runs with under 80% accuracy were excluded. Also, five subjects were excluded from the analysis of Experiment 1A because their average accuracies were under 60%. Because of the increased signal-to-noise ratio, the first three trials were also removed from the beginning of each run. This only affects the visualization since the first presentations of stimuli (12 trials) were not used for statistical analysis. According to these criteria, 9.5% of all trials were excluded in Experiment 1A (0.3% of trials with RTs greater than mean + 3 *SD*, 0.1% trials with RT shorter than 200 ms, and 9.1% trials in runs with accuracy under 80%).

#### Statistics

We analyzed three conditions (P1, P2, and S denoting the first image in the pairs, the second image in the pairs, and the single stimuli, respectively) based on their position in the pattern. Two conditions (P1 and P2) are related to learning. Stimuli positioned as the first events (P1) of the associated pairs have a function of anticipation. Thereby the second events (P2) of the stimulus pairs become predictable. Stimuli positioned as the second events (P2) represent a priming effect. The condition of the single stimuli (S) serves as a reference, as it does not have a role in the learning. Although the difference in RT or accuracy between P1 and P2 may reflect mixed effects of priming and anticipation, the summation of these two effects could be an even more sensitive index of VSL. For statistical analysis, the first presentation of the chunks was removed from each run since no learning effect is possible.

Mean RTs for P1, P2, and S were compared among runs within subjects by one-way repeated-measures ANOVA followed by Tukey-Kramer post hoc tests for pairwise comparisons, while median accuracies were compared by the Friedman and post hoc Wilcoxon signed-rank tests.

### Results and discussion

After the evaluation of the responses for the interview questions of the participant, we found only one participant who reported suspecting some pattern in stimulus presentation order according to post-test interview questions (*Task and procedure* section). However, we decided not to exclude these data from the analysis as the participant could not recall specific examples of image pairs.

**Fig. 3:**
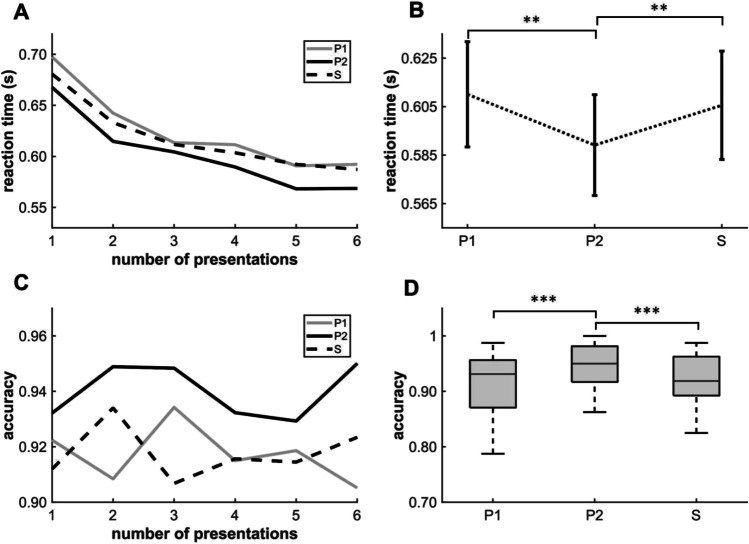
Performance on Experiment 1A. (**A**) Changes in P1, P2, and S reaction times (y-axis) within runs as a function of presentation number (x-axis). (**B**) Comparisons of mean reaction times among conditions ((F(2,64) = 10.002, p < 0.001 by repeated-measures ANOVA) (*mean* P1 = 0.614 s, *SD* = 0.133 s; *mean* P2 = 0.592 s, *SD* = 0.123 s; *mean* S = 0.611 s, *SD* = 0.133 s.). (**C**) Changes in median P1, P2, and S accuracy (y-axis) as a function of presentation number (x-axis). (**D**) Comparisons of median [interquartile range (IQR)] accuracies (n = 33, χ^2^ = 20.37, p < 0.001 by the Friedman test) (P1: *median* (*MED)* = 0.931, *IQR =* 0.085; P2: *MED* = 0.95, *IQR* = 0.064; S: *MED* = 0.919, *IQR* = 0.07, ** p < 0.01 and *** p < 0.001 by post hoc pairwise comparisons). The error bars in B indicate the standard error of the mean, while those in D indicate the IQR

In line with the results of Turk-Browne et al. (2010), RT data revealed a strong learning effect (F(2,64) = 10.002, *p* < 0.001) (Fig. 3B). Specifically, pairwise comparison showed a significant priming effect (lower mean RT) for categorization of P2 (*mean =* 0.592 s, *SD* = 0.123 s) compared to categorization of S (*mean =* 0.611 s, *SD* = 0.133 s; *q* = −3.484, *p* = 0.004, Cohen’s *d* = 0.148). There was also a significant difference in mean RT between conditions P1 (*mean =* 0.614 s, *SD* = 0.133 s) and P2 (*q* = 3.94, *p* = 0.001). A post hoc power analysis was performed (Monte Carlo simulation with 1,000 iterations), and a power of 98.7% was obtained. In contrast to the original findings of Turk-Browne et al. (2010), there was no significant difference in RT between P1 and S, indicating no detectable effect of anticipation.

As the difficulty of the task was increased compared to the original task of Turk-Browne et al. (2010), it was also sufficiently sensitive to show a learning effect on categorization accuracy (*n* = 33, *χ*^2^ = 20.33, *p* < 0.001 by the Friedman test) (Fig. 3D). Pairwise comparisons revealed differences in median accuracy between condition P2 (median *(MED) =* 0.95, interquartile range (*IQR*) *=* 0.063) and S (*MED =* 0.919, *IQR =* 0.070) (*z* = -3.806, *p* < 0.001, *r* = 0.663) and between P1 (*MED =* 0.931, *IQR =* 0.086) and P2 (*z* = -3.45, *p* < 0.001). In the case of the accuracy data, the post hoc power analysis (with the same parameters as previously) showed a power of 99.0%.

Therefore, using a task modified from the original, we demonstrated that the first image in pairs (P1) primed categorization of the second image (P2), thereby demonstrating a robust VSL. This priming effect was manifested by both shorter P2 RTs and greater categorization accuracy. However, there was a possible confound in this experiment, which we already indicated in the *Introduction*. The categories in all pairs were category-alternating (Small–Large or Large–Small), therefore there were more category-alterations than repetitions of consecutive stimulus categories in the sequence. This more frequent alternation between the responses could result in procedural learning of alternating key pressings. Consequently, the faster and more accurate responses to the P2 condition might only reflect that it always required an alternating response, which was much more frequent in the experiment.

In an attempt to identify a confounding effect of motor learning, we ran an additional analysis where the category-repeating trials had been excluded. Hypothetically, the responses in these trials were slower than in the other trials, because category-repeating trials were rare in the sessions. These occurred only at junctions of unpaired stimuli and stimulus pairs, but not within pairs, which means that the excluded trials contained stimuli only from the P1 and S conditions.

With this and the above-mentioned criteria, we lost 38% of all trials. After removal, neither mean RT nor median accuracy differed among conditions (P1 *mean* RT = 0.595 s, *SD* = 0.112; P2 *mean* RT = 0.592 s, *SD* = 0.119; S *mean* RT = 0.594 s, *SD* = 0.132; *F*(2,64) = 0.145, *p* = 0.866; P1 *MED* accuracy = 0.942, *IQR*: 0.084; P2 *MED* accuracy = 0.95, *IQR*: 0.064; S *MED* accuracy = 0.94, IQR: 0.057; n = 33, *χ*^2^ = 0.14, *p* = 0.934).

Thus, this reanalysis excluding category-repeating trials suggests that the observed priming effect is the product of motor learning. Further, the effect of this motor learning confound appeared to increase from run to run independently from the changed stimuli set, as the difference between conditions already appears at the beginning of the runs (Fig. 3A, C). Therefore, the design is not appropriate for investigating the VSL trajectory. In the next experiment, we investigated whether the previously observed priming effect reflects the combination of VSL and motor learning using a modified sequential categorization task including both category-alternating and category-repeating pairs (Fig. 2C).

## Experiment 1B

To verify our presumption that motor learning contributed to the results of Experiment 1A, the regularity of the image sequence was modified in Experiment 1B by equalizing the numbers of category-alternating and category-repeating pairs. All other parameters of the design and the task remained the same as in Experiment 1A.

### Material and methods

#### Participants

Thirty-eight healthy right-handed volunteers with normal or corrected-to-normal vision participated in Experiment 1B (18 females, mean age: 27.6 years; range: 21–42 years). All gave written informed consent and the protocol was approved by the Human Investigation Review Board of University of Szeged. Three subjects were excluded from analysis because their average accuracies were under 60%. Based on the exclusion criteria described for Experiment 1A (*Data processing*), we excluded overall 2.61% of all trials in Experiment 1B (0.8% of trials due to RT > mean + 3 *SD*, 0.02% due to RT < 200 ms, and 1.8% of trials in runs with accuracy < 80%).

#### Pattern

Similar to Experiment 1A, the stimuli were grouped into four associated pairs, but only two were category-alternating (Large–Small and Small–Large) while the other two were category-repeating (Large–Large and Small–Small). The unpaired stimuli included two Large and two Small object images as before (Fig. 2C). Using this combination of stimuli, the ratio of different transitions between categories was close to 1:1 (47.6% probability of category repetition within trials). Therefore, the transitions of successive categories could not create a motor pattern biasing the VSL process. Other parameters (exposition time, ITI) were the same as in Experiment 1A.

### Results and discussion

One participant reported suspecting regularity but the data were retained for the same reason as in Experiment 1A.

**Fig. 4:**
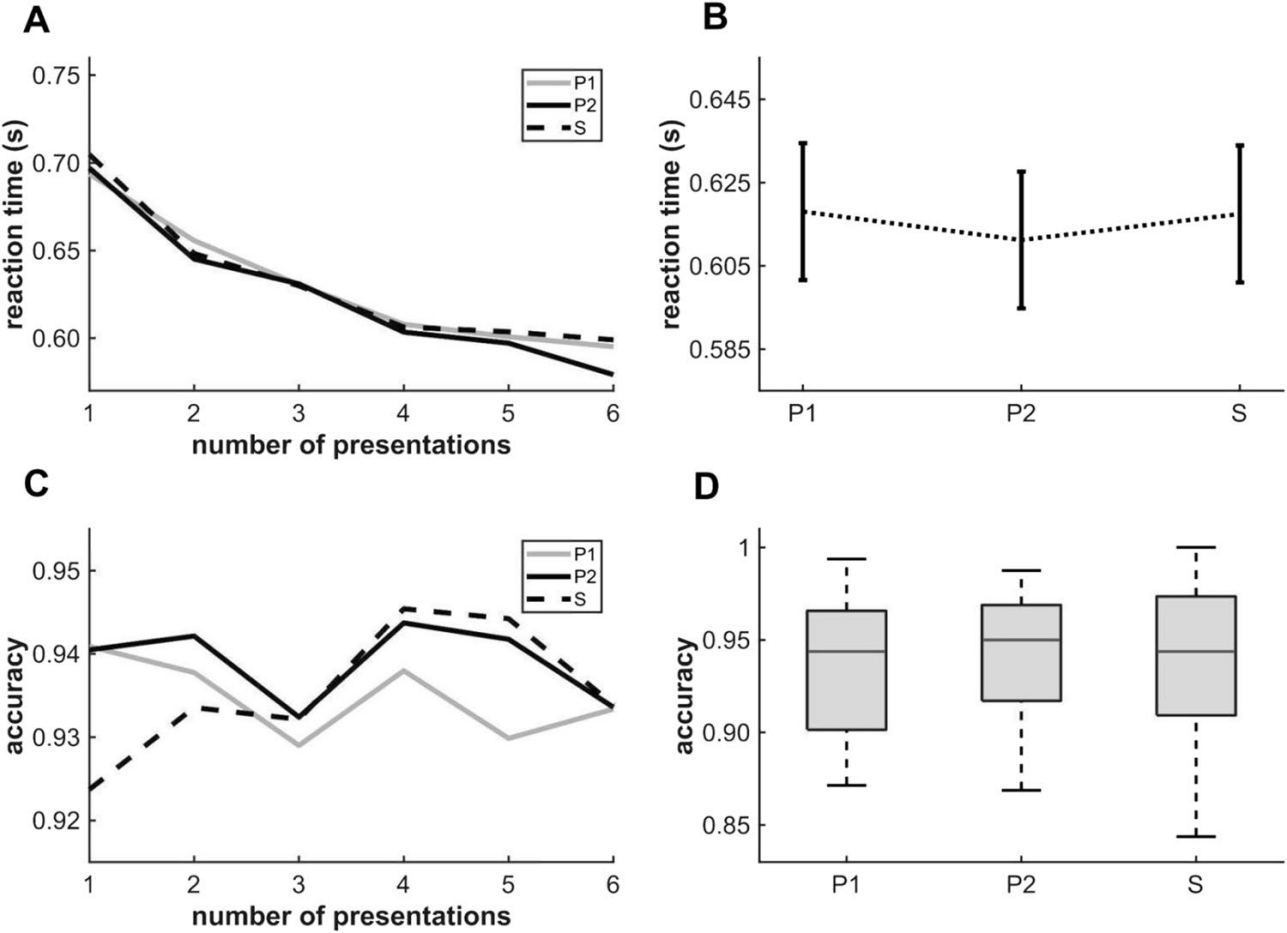
Performance in Experiment 1B. (**A**) Changes in P1, P2, and S reaction times (y-axis) as a function of presentation number (x-axis). (**B**) Comparison of mean reaction times (*F*(2,68) = 2.458, *p* = 0.093 by repeated-measures ANOVA) (P2: *mean* = 0.611 s, SD: 0.097 s; S: *mean* = 0.617 s, *SD* = 0.097 s; P1: *mean* = 0.618 s, *SD* = 0.097 s). Error bars indicate the standard error of the mean. (**C**) Median accuracies for categorizing P1, P2, and S (y-axis) as a function of presentation number (x-axis). (**D**) Comparison of median accuracies (n = 35, *χ*^2^ = 0.41, *p* = 0.814) by the Friedman test (P1: *median (MED)* = 0.944, *interquartile range (IQR)* = 0.064; P2: *MED* = 0.95, *IQR* = 0.052; S: *MED* = 0.944, *IQR* = 0.064). The error bars indicate the IQR

A one-way repeated-measure ANOVA revealed a tendency in RT among conditions (Fig. 4A, B) (*F*(2,68) = 2.458, *p* = 0.093; P2: *mean* = 0.611 s, *SD* = 0.097 s; S: *mean* = 0.617 s, *SD* = 0.097 s; P1: *mean* = 0.618 s, *SD* = 0.097 s), while there were no differences in median accuracy according to the Friedman test (Fig. 4C, D) and pairwise comparisons (P1: *MED* = 0.944, IQR: 0.064, P2: *MED* = 0.95, IQR: 0.052; S: *MED* = 0.944, IQR: 0.064). Since neither VSL markers were statistically significant, we did not investigate learning trajectories.

### Comparing the results between experiments 1A to 1B

Experiment 1B replicates the design of Experiment 1A but with modification of the regularity in image category sequence. Adding category-repeating pairs roughly balanced the category transitions, thereby eliminating the possibility of motor or procedural learning. As expected from a substantial contribution of motor or procedural learning to the results of Experiment 1A, this modification largely eliminated the differences in RT and accuracy among conditions (the anticipation and priming effects) despite doubling the subject sample size compared to the original study of Turk-Browne et al. (2010)).

**Fig. 5:**
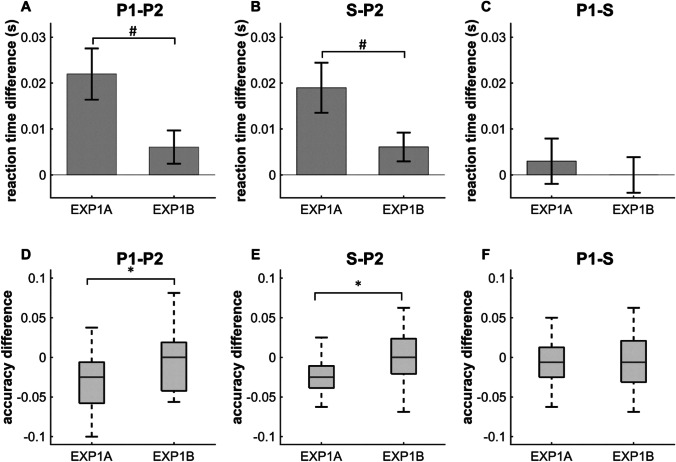
Performance differences between Experiments 1A and 1B indicating a contribution of procedural or motor learning to the learning trajectories in Experiment 1A. (**A**) The difference in reaction time between conditions P1 and P2 (P1-P2) was tendentiously greater in Experiment 1A than in Experiment 1B (*mean* = 0.022 s, *SD* = 0.032 s vs. *mean* = 0.006 s, *SD* = 0.021 s). (**B**) The difference in reaction time between conditions S and P2 (S-P2) was also tendentiously greater in Experiment 1A than in Experiment 1B (*mean* = 0.019 s, *SD* = 0.031 s vs. *mean* = 0.006 s, *SD* = 0.019 s). (**C**) The difference in reaction time between P1 and S (P1-S) was not altered by the experimental modifications. (**D**) The difference in median accuracy between P1 and P2 (P1-P2) was significantly greater in Experiment 1A than in Experiment1B (*median (MED) =* −0.025, *interquartile range (IQR) =* 0.052 vs. *MED* = 0.0, *IQR* = 0.061. (**E**) The difference in median accuracy between P2 and S (P2-S) was also significantly greater in Experiment 1A (*MED* = −0.025, *IQR* = 0.027 vs. *MED* = 0.0, *IQR* = 0.044. (**F**) The difference in median accuracy between P1 and S was similar for both experiments. Error bars from A–C indicate the SEM and those from D–F indicate the IQR (*p < 0.05 and #*p* < 0.1)

A direct comparison between the results of Experiments 1A and 1B (Fig. 5) revealed a tendentiously higher priming effect in Experiment 1A as evidenced by the greater difference in mean P2 and S (P2-S) RTs than in Experiment 1B (*mean* = 0.019 s, *SD* = 0.031 s vs. *mean =* 0.006 s, *SD* = 0.019 s, *t* = 2.083, df = 66, *p* = 0.082 by t-test with Holm–Bonferroni correction). Further, the difference in median accuracy between P2 and S was also significantly greater in Experiment 1A than in Experiment 1B (*MED* =−0.025, *IQR =* 0.028 vs. *MED* = 0.0, *IQR* = 0.044, *p* = 0.0315 by the Mann–Whitney U-test with Holm–Bonferroni correction) (Fig. 5B, E). The difference between mean P1 and P2 RTs (P1-P2) was also tendentiously higher in Experiment 1A than in Experiment 1B (*mean* = 0.022 s, *SD* = 0.032 s vs. *mean* = 0.006 s, *SD* = 0.021 s; *t* = 2.421, df = 66, *p* = 0.054 by t-test with Holm–Bonferroni correction). In addition, the difference in median accuracy was significantly greater in Experiment 1A than in Experiment 1B (*MED* = −0.025, *IQR =* 0.052 vs. *MED* = 0.0, *IQR* = 0.061, *p* = 0.039 by the Mann–Whitney U-test with Holm–Bonferroni correction) (Fig. 5A, D). These differences can be explained by additional procedural or motor learning in Experiment 1A. There were no significant differences or trends between Conditions P1 and S (Fig. 5C, F).

In the additional analysis of the category alternating pairs in Experiment 1A, the significance of SL effect was lost. The effect sizes and differences in mean RT and median accuracy among conditions in Experiment 1A were reduced when category transitions were balanced. These results seen in the additional analysis of Experiment 1A and the analysis of Experiment 1B and their comparison imply that the learning observed in Experiment 1A was most likely due to priming of the motor response pattern rather than VSL.

In this relatively difficult task in Experiment 1B, we did not find any sign of learning in the accuracy data. Additionally, some participants reported categorization difficulties with certain pictures, which may have further reduced differences in RT and accuracy among conditions (i.e., markers of learning) by introducing chance trials. Nonetheless, the mean P1-P2 RT was nonzero in Experiment 1B, suggesting some degree of VSL. Therefore, we repeated these measurements with additional task modifications aimed at increasing the effect sizes of such differences.

## Experiment 2

After we eliminated the motor learning effect by balancing the category transitions, the design in Experiment 1B showed a drastically smaller effect in the two markers of VSL compared to Experiment 1A. Hence our next goal was to demonstrate priming and/or anticipation effects and thus establish the learning trajectory across runs. To this end, the following modifications were made to the protocol used in Experiment 1B: (1) we simplified the discrimination task, (2) we increased the number of control, single stimuli, (3) we raised the number of subjects and presentations, (4) we added a random initial phase at the beginning of each run, and (5) we applied a random ITI.

1. The rationale behind using the difficult task in Experiments 1A and 1B is to increase the sensitivity of the design for the learning effect. However, since the accuracy was not sensitive enough for the paradigm, we did not continue to use the difficult task in Experiment 2. Moreover, using easier tasks is expected to increase the sensitivity of the RT data for learning.
2. The transition probabilities between stimuli within pairs were always 100% in the protocol used for Experiments 1A and 1B, while the transition probabilities for other cases were 14.3%. In Experiment 2, the number of single stimuli was increased from four to eight to reduce the transition probabilities between the second image of a pair and a single image to 12.5% and that between any two single images to 9.1%. Adding more single images thus increased the contrast between the transition probabilities of the first and second images in pairs versus the transition probabilities for all other stimulus transitions. This change may also decrease the speed of learning by increasing the temporal distance among associated stimulus pairs.
3. To increase the chances of acquiring sufficient data to detect learning across runs, we further increased the repetition number of the information chunks. In addition to potentially increasing the effect size, this change could also reduce the speed of learning and enhance the temporal resolution (if behavioral changes are observed across a greater number of presentations). However, slower learning would reduce the expected change in RT over a given repetition number, so we also raised the number of subjects to increase the statistical power.
4. The most critical period of the measurement is the first few presentations when the naïve participants face the procedure and the stimuli for the first time. Without routine, the data from this period can contain noise that can alter the learning curve. The procedural learning effect and task familiarization (Manahova et al., 2019) are indicated by a quick drop in RT at the start of each run. Therefore, we tried to eliminate this skewing effect by inserting an additional warm-up period at the beginning of each run.
5. Random ITI was used to reduce the monotony of the task, which may help sustain attention on the images.

### Method

#### Participants

Eighty-seven healthy right-handed volunteers with normal or corrected-to-normal vision participated in Experiment 2 (48 females, mean age: 21.26 years; range: 18–28 years). All provided written informed consent and the protocol was approved by the Human Investigation Review Board of University of Szeged. Four subjects were excluded from the analysis because their average accuracies were under 60%.

In Experiment 2, the same exclusion criteria were used as in the previous experiments, except that we excluded RT measures above mean + 2 *SD*. The reason behind this change was due to the inclusion of the warm-up period, which eliminated the sudden RT drop during the early phase of each run and reduced the overall variance (allowing a stricter criterion, see further details in the Online Supplementary Material (OSM)). Overall, we excluded 4.15% of all trials in Experiment 2 (2.1% of trials due to RT > mean + 2 *SD*, 0.2% due to RT < 200 ms, and 1.8% of trials in runs with accuracy under 80%).

#### Stimuli

Eight complex images of everyday objects and eight images of animals were previously selected for each run from the BOSS (Brodeur et al., 2010; Brodeur et al., 2014).

#### Design

A stream of visual stimuli was presented to each participant and responses and RTs were measured across two runs. For one run, eight-eight randomly chosen images were used from both categories of the stimulus set. The same stimuli were not presented again in the second run. The number of presented stimuli was increased from 12 to 16 per run. The randomly chosen 16 novel stimuli were used to create a temporal sequence specific for each run. A warm-up period was also inserted at the beginning of each run where the same 16 stimuli were presented ten times in completely random order. After this period, the presentation of the images was continued without any cue, but this time they formed information chunks as single stimuli or associated stimulus pairs in a similar way to the way they did in the previous experiments. The information chunks were presented 15 times in a pseudo-random order. Each run contained a sequence of 400 trials (160 random, 240 structured).

#### Task and procedure

The task used a 2AFC design in which subjects had to decide whether the image was of an object by pressing button 1 with the right index finger or an animal by pressing button 2 with the right middle finger on a numeric keyboard with no feedback. After their response, a jittered ITI was applied (500–1,200 ms). Participants were seated approximately 60 cm from a desktop computer screen (resolution 1,920 × 1,080 pixels, 23-in., 60 fps), and all were naïve to the pattern of the stimulus stream. After each measurement, the participants were asked the same five questions regarding recognition of patterns as in Experiment 1 to determine if learning had become explicit, using the same questions we described in Experiment 1A.

#### Pattern

**Fig. 6:**
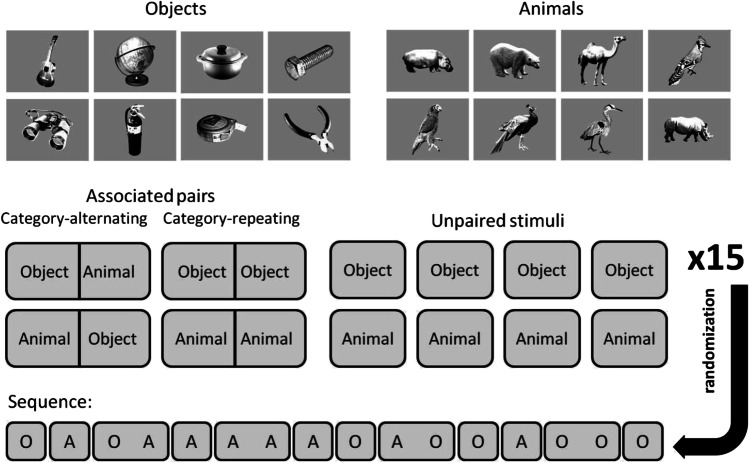
Examples of stimuli from the two categories Object (O) and Animal (A) used in Experiment 2. Eight of the 16 pictures were used to create four stimulus pairs (Animal–Object, A O; Object–Animal, O A; Animal–Animal A A; Object–Object, O O) as in Experiment 1B. The remaining eight stimuli were unpaired stimuli (condition S)

The pattern was similar to that used in Experiment 1B, but the number of stimuli in the single condition was increased from four to eight and each stimulus pair was followed by at least one unpaired stimulus. In other aspects, the regularity was the same (Fig. 6). Thus, each sequence contained two associated pairs with alternating categories (Animal–Object and Object–Animal) and two pairs with repeating categories (Animal–Animal and Object–Object). The remaining four-four images of objects and animals were presented as single stimuli.

#### Statistics

To evaluate the RT data, we used a linear mixed-effect model with restricted maximum likelihood criterion. The model was fit to the data using the lme4 package in R (Bates et al., 2014). The linear mixed-effect model was chosen instead of a generalized linear mixed-effect model based on residual diagnostics (for further details, see OSM) and the robustness of the linear mixed-effect model. The model included the natural logarithm of repetition number and condition (P1, P2, and S) as fixed effects and the log (repetition number) × condition interaction. The natural logarithm of repetition number was used because reduced model (without the random slope) including this measure had a smaller Akaike information criterion value than the non-logarithmic repetition model (−58674 and −58672, respectively). Also, the non-logarithmic repetition model with added random slope for repetition failed to converge.

In addition, the jittered ITI was added as a fixed effect. For random effects, we included random intercept and slope terms for subjects, a random intercept for the different pictures, and a random intercept for a new variable NVAR ranging between 1 and 4 based on current and previous answers because the proper processing of the preceding stimulus is assumed to be crucial in the paradigm. The trial was scored “NVAR 1” if current and previous answers were both correct, “NVAR 2” if the current answer was correct but the preceding answer was incorrect, “NVAR 3” if the preceding answer was correct but the current answer was incorrect, and “NVAR 4” if both were incorrect.

The random effects were evaluated with the likelihood ratio test (lmtest package, models refit with maximal likelihood criterion (Zeileis & Hothorn, 2002)) using a restricted model without the given random effect. For appraisal of fixed effects, type III ANOVA was used from the lmerTest package (Kuznetsova et al., 2017). To obtain p values, we used Satterthwaite's method for the calculation of degrees of freedom.

If ANOVA revealed a significant effect, we conducted pairwise comparisons using the z test. For fixed effects, estimated marginal means were used (emmeans package (Lenth et al., 2021)) and the p values were adjusted according to Tukey. For the interaction, we used estimated marginal means of linear trends (emmeans package) with the natural logarithm of repetition number as the linear predictor. Again, p values were adjusted according to Tukey.

For the accuracy data, a generalized linear mixed-effect model was fit with binomial distribution (lme4 package) because the only possible responses were correct (1) and incorrect (0). The fixed effects were then tested with type III Wald χ^2^ test (using the lmerTest package (Kuznetsova et al., 2017)). The model included the natural logarithm of repetition number, condition, their interaction, and ITI. The subject number and identity of the picture were added as random effects.

In case of a significant result, we performed a post hoc power analysis using the simr package (Green & MacLeod, 2016).

### Results and discussion

**Fig. 7:**
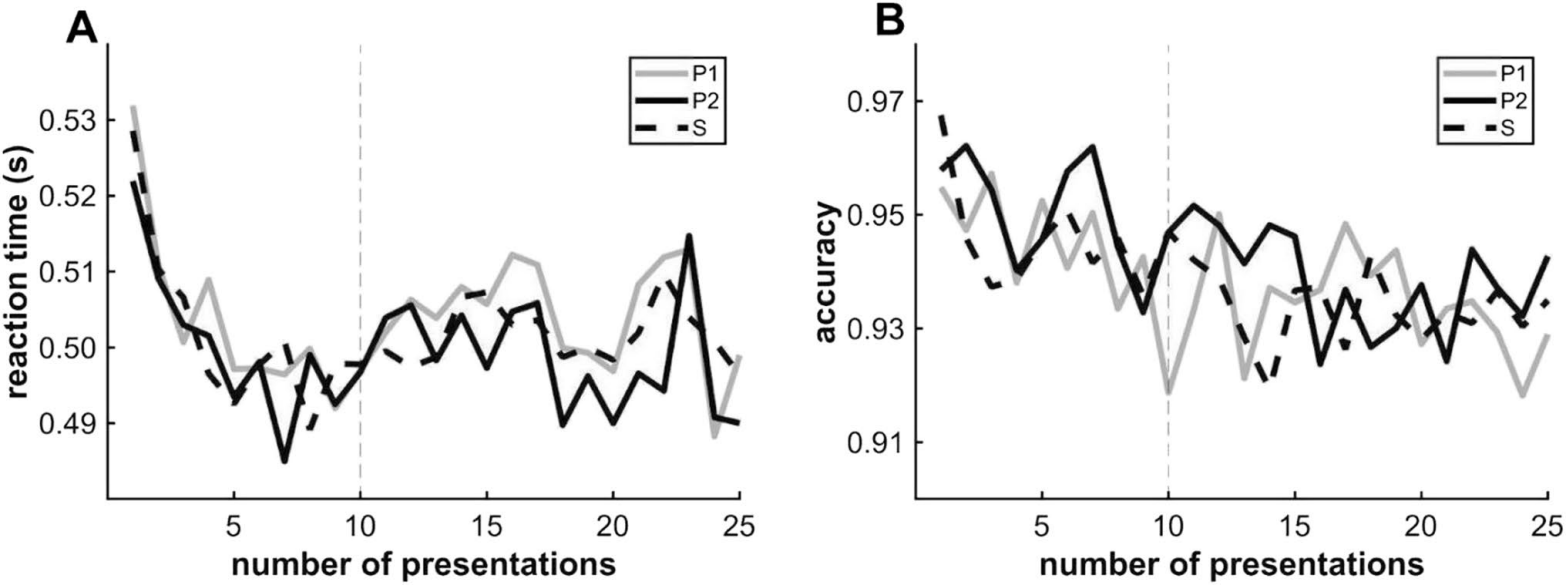
Reaction times and response accuracies in Experiment 2. (**A**) Average reaction time (y-axis) as a function of repetition number for the three conditions P1, P2, and S. (**B**) Average accuracy (y-axis) as a function of repetition number for the three conditions. The gray vertical dotted lines mark the repetition number at which regularities started (i.e., after the random sequence warm-up period from presentation numbers 1 to 10)

**Fig. 8:**
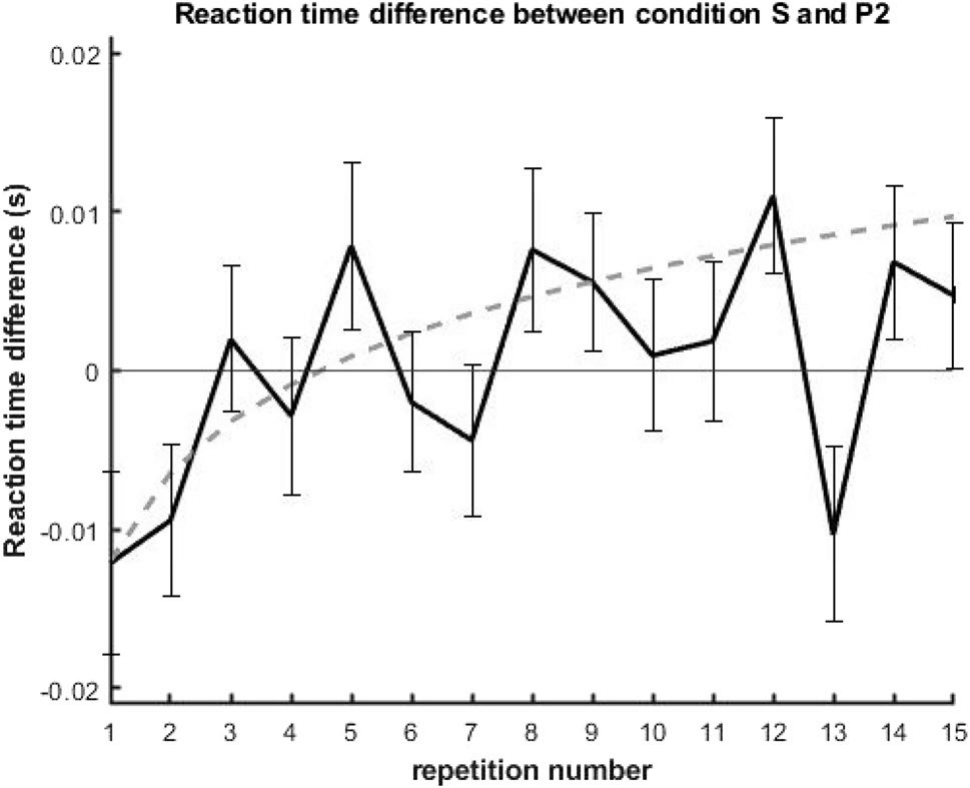
Learning trajectory in Experiment 2 as indicated by the change in reaction time difference between conditions S and P2 (S-P2) (y-axis) as a function of repetition number (x-axis)

No participant reported explicit knowledge of regularity, and as expected, the participants could easily perform the task with high accuracy. Thus, the ceiling effect diminished the sensitivity of accuracy data for the learning effect with this simpler task. (Fig. 7B). Neither the fixed effects nor their interactions showed a significant difference in the responses except for the length of the jittered ITI (repetition: *χ*^2^ = 1.9488, df = 1, *p* = 0.163; condition: *χ*^2^ = 2.3084, df = 2, *p* = 0.315; interaction: *χ*^2^ = 1.2366, df = 2, *p* = 0.539; ITI: *χ*^2^ = 14.9357, df = 1, *p* < 0.001). Since neither of the expected fixed effects nor the interaction was significant, we conducted no further analysis of error rates. (Figure 8)

In contrast to accuracy, there was a significant log(repetition number) × condition interaction effect on RT (Table 1, Fig. 7A). Similar to accuracy, there was also a significant fixed effect of ITI (Fig. 7A, Table 1).

**Table 1:** Results of ANOVA for fixed effects in the linear mixed-effect model

	Degrees of freedom (Satterthwaite’s method)	*F* value	*p* value
Repetition	1, 85	0.178	0.674
Condition	2, 38030	3.344	0.035
Intertrial interval	1, 38048	407.456	<0.001
Interaction	2, 38018	4.303	0.014

The pairwise comparison of the estimated marginal means (Table 2A) under the different conditions were inconclusive as none reached significance (Table 2C). However, post hoc testing of the interaction was more satisfactory. Comparison of the linear trends (Table 2B) under the three conditions revealed that the slopes for S and P2 differed significantly, while P1 and P2 slopes showed a tendentious difference (Table 2D).

**Table 2:** Evaluation summary of the linear mixed effects model for RT

**A**	EMM (s)	SE	**C**	*z* value	*p* value
**S**	0.463	0.0281	**S-P1**	−1.804	0.168
**P1**	0.465	0.0282	**S-P2**	0.158	0.986
**P2**	0.462	0.0282	**P1-P2**	1.701	0.205
**B**	EMM trend	SE	**D**	*z* value	*p* value
**S**	0.00117	0.0002	**S-P1**	0.320	0.945
**P1**	0.00058	0.0023	**S-P2**	2.856	0.012
**P2**	−0.00408	0.0023	**P1-P2**	2.195	0.072

Estimated marginal means (EMM) of the different conditions (**A**) and of the linear trends (EMM trend) in the different conditions (**B**) with the presentation number as the linear predictor. Pairwise comparisons of the estimated marginal means (**C**) and linear trends (**D**) between conditions

We performed a post hoc power analysis using 1,000 iterations to test how many times a difference in the interaction of the fixed effects would emerge. Using the same statistical methods as described above (type III ANOVA and Satterthwaite’s method) yielded 76% observed power.

The trends of the S and P2 RT curves (representing the priming effect) differed significantly in the linear mixed model, suggesting VSL, so we analyzed the change in RT difference (S-P2) across repetitions. The S-P2 RT difference as a function of repetition numbers was fit to a linear regression model and used to test the hypothesis that learning progressed logarithmically. For this analysis, we fit two model variants to the data, one with a linear predictor (model a: RT difference by repetition number) and one with a logarithmic predictor [model b: RT difference by log(repetition number)]. Both models reached significance (model a, *R*^2^ = 0.005, *F*(1,1243) = 6.296, *p* = 0. 012; model b, *R*^2^ = 0.008, *F*(1,1243) = 9.767, *p* = 0.002), so to evaluate the logarithmic nature, we performed an encompassing test using the encomptest() function in R (part of the lmtest package (Zeileis & Hothorn, 2002)). This encompassing test indicates whether a new model combining model a and b provides additional information compared to the original models (hence reaching significance). We also found that model b with the logarithmic scale was a better fit to the combined model (model a vs. encompassed model: *F*(−1, 1242) = 4.984, *p* = 0.026, model b vs. encompassed model: *F*(−1, 1242) = 1.528, *p* = 0.217), as comparing the encompassed model to model b did not yield additional information.

### General discussion

The primary goal of the present study was to develop a task design for investigating unsupervised implicit VSL online, thereby both validating behavioral correlates of VSL markers (priming and anticipation) and revealing the dynamic properties of the learning process. A modified version of the task developed by Turk-Browne et al. (2010) with counterbalanced category transitions and a greater number of control images yielded a significant change in the priming effect on the second image (P2) in a pair across repetitions as measured by RT. Furthermore, this effect could not be explained by procedural learning. The change in priming across repetitions was best fit by a model including the logarithm of repetition number, suggesting that VSL is a relatively rapid process.

Priming refers to the facilitated recognition or classification of the second stimulus in a pair by the first stimulus, manifesting as faster RT or greater accuracy. Anticipation, on the other hand, influences processing of the first image in an associated pair because it predicts the characteristics of the second. We adapted and modified the paradigm of Turk-Browne et al. (2010) and replicated their results showing priming of the second image in category-alternating pairs as evidenced by reduced P2 RT. These modifications included increasing the number of test subjects along with the number of runs for statistical power, and using a more challenging discrimination task to increase the sensitivity to VSL. In accordance with the original study, this paradigm (Experiment 1A) revealed a priming effect on P2 RT but no sign of anticipation. A priming effect was also found in the accuracy data. However, reanalysis suggested that motor and procedural learning accounted for these results.

Therefore, we examined the possible contributions of motor learning using a task with additional modifications including the addition of category-repeating pairs (images from the same category) in Experiment 1B. The findings of Experiment 1B confirmed the contribution of motor learning in Experiment 1A but also drastically reduced effects on VSL markers. We therefore modified the task again to increase the sensitivity of the priming effect.

Subsequently, we aimed to modify our paradigm in several aspects to achieve a sufficient amount of data to prove the priming and/or anticipation effects as well as to investigate the learning progression: (1) we simplified the discrimination task, (2) we increased the number of control single stimuli, (3) we raised the number of subjects and presentations, (4) we added a random initial phase in the beginning of the run, and (5) we applied a random ITI. With these modifications, we achieved our goal to find a model fitting of the learning trajectory based on RT priming revealed that VSL is progressive and relatively rapid.

#### Statistical and motor learning

Many researchers have argued that in SRT tasks the presumed behavioral correlates of implicit learning was the result of motor learning instead of perceptual learning (Lungu et al., 2004; Willingham et al., 1989; Ziessler, 1994). While it has been shown that implicit perceptual and sequence learning can occur in the absence of motor response patterns (Heyes & Foster, 2002; Mayr, 1996; Robertson & Pascual-Leone, 2001), Dennis et al. (2006) have stated that in many cases these paradigms failed to completely eliminate motor learning.

Driven by the presumption that the adopted regularity resulted in a pattern of motor responses, the structure of the information chunks was modified in Experiment 1B. In the original paradigm the associated pairs only contained category-alternating stimuli. This higher-level regularity resulted in a shortened RT in case of a category alternating response and impaired the RT of those stimuli that were repeating categories, which are the non-predictable stimuli (first member of the associated stimulus pair and the control, single stimuli). In Experiment 1B, we added stimulus pairs that were category-repeating, thus the category alternations and repetitions were balanced out among the responses and among the second members of the associated stimulus pairs, hence we were able to eliminate the motor learning confound.

#### Temporal dynamics of implicit VSL

Beyond the studies from the domain of language acquisition using online measurement, there is a similar investigation from Siegelman and his colleagues (Siegelman et al., 2018). They described the learning curve of VSL with an explicit self-paced paradigm involving explicit learning as the participants were informed about stimulus regularity and actively searched for patterns in the stimulus sequence. By contrast, our results revealed the dynamics of implicit VSL as only one participant in each of Experiments 1A and 1B reported some explicit knowledge of stimulus regularity, while in Experiment 2, all participants remained naïve to the regularity even at the end of the measurement.

In Experiment 2, we found that a linear mixed-effect model fit the learning trajectory and the progression of the priming effect (linear trend) differed significantly between P2 and S. Thus, this marker develops during the continuous presentation of an environmental regularity and reduces the RT to a predictable stimulus. In contrast, we found no behavioral correlates of the anticipatory effect or associations with SL.

Further, the trajectory was best fit by a logarithmic model, indicating that this form of VSL emerges rapidly during stimulus presentation. This finding is in line with the results of Siegelman and colleagues, although again learning in their case had an explicit component. This modeling of the temporal dynamics of VSL will be essential for future studies investigating the underlying neural correlates. By modeling the learning trajectory, we can determine not only the final result of the learning but the different parts of the learning process, and with this knowledge, we can tie the neural data to segmented parts of the learning. This way determining crucial areas and function of the central nervous system related to VSL can be achieved.

#### Limitations and future directions

A major limitation of this study is that in the second experiment many changes have been made in one step compared to the first paradigm (Exps. 1A and 1B). Although the effect of each modification cannot be proven at this point, they can be deduced. The most important change was an increase in the number of repetitions. This is the only change that actually helps to achieve a greater effect. Using a higher number of single trials increases the transitional probabilities between P2-S and S-P1, thus the contrast is greater. However, this change did not increase the RT reduction for condition P2. Also, using an easier task and a warm-up period reduced the variance in RT. Future studies are needed to verify the inferences of these changes.

Using two markers, our design is suitable to investigate the priming and anticipatory effect separately as two of the components of SL. We could not detect any anticipatory effect, even though the perception of the first stimuli is supposedly affected by learning. A future aim is to develop a new design with which we can create a more optimized model that can describe SL and eventually can be sensitive enough to show an anticipatory effect. The ability to observe the two markers separately could yield additional information about SL.

### Conclusion

We have developed a viable online tool to measure the dynamic behavioral correlates of unsupervised implicit VSL. We applied an SRT method with associated image pairs and single images investigating two VSL markers: priming of the second, predictable image in a pair and anticipation of the first image.

First, we assessed a paradigm adapted for a previous study that seemed to be confounded by motor learning, which was tested and the motor confound was confirmed and excluded. Second, using an improved novel paradigm, linear mixed-effect modeling, and estimated marginal means of linear trends, we measured implicit VSL, specifically the priming effect on predictable stimuli. The learning curve fitted a logarithmic model, suggesting a rapid learning process.

## Supplementary Information

ESM 1 (DOCX 171 kb)
