# Supplementary material for: Online measurement of learning temporal statistical structure in categorization tasks
Source: Mem Cognit. 2022 Apr 4;50(7):1530–45. doi: 10.3758/s13421-022-01302-5 (PMC9508059; doi:10.3758/s13421-022-01302-5)
Supplement: Supplementary file 1 — (DOCX 171 kb) [file 13421_2022_1302_MOESM1_ESM.docx]

**10. Supplementary material**

**Using parametric test on reaction time data**


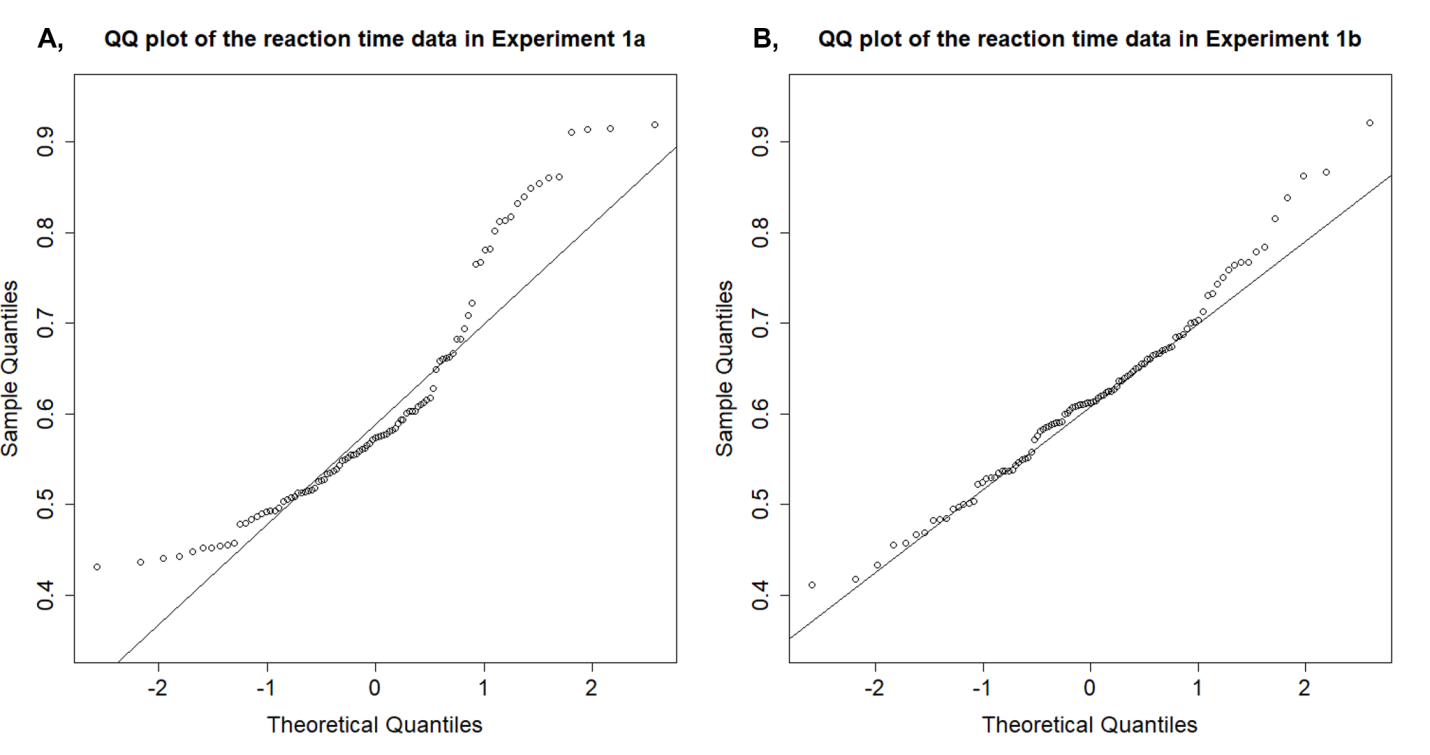


**Supplementary fig. 1** A, Quantile–Quantile (QQ) plot of the residuals from Experiment 1A.

B, Quantile–Quantile (QQ) plot of the residuals from Experiment 1B. The distributions of RT in Experiment 1A and 1B are close to normal, and due to the robustness of ANOVA, this analysis is applicable to our data.


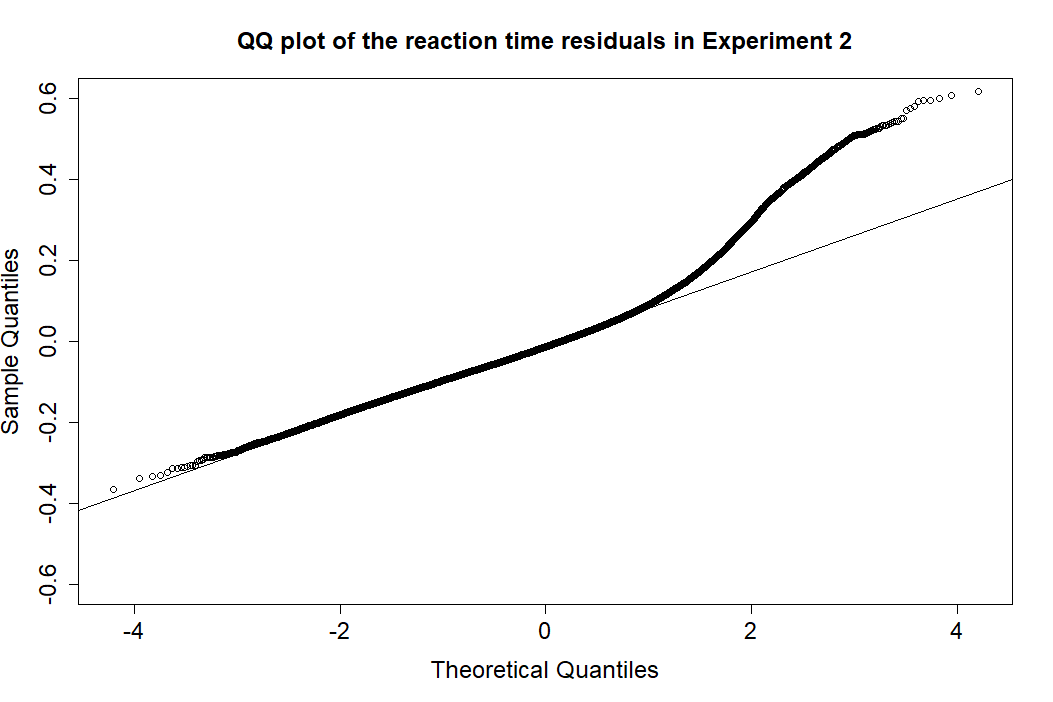


**Supplementary fig. 2** Quantile–Quantile (QQ) plot of the residuals from Experiment 2. Based on the distribution of the residuals, a linear mixed effect model was used instead of a generalized linear mixed effect model.

**Data exclusion criteria**

We chose to exclude RTs slower than mean + 3 × *SD* in Experiments 1A and 1B because these paradigms were strongly related to those used in the study of Turk-Brown et al. (2010), so we followed their methodological approach. However, in Experiment 2, many parameters were changed. The warm-up period reduced the rapid RT drop at the beginning of the blocks, thereby reducing the overall variance in RT. Therefore, we used stricter exclusion criterion mean + 2 × *SD*. The paradigm works with 3 *SD* as well:

**Supplementary table 1** Evaluation of fixed effects in the linear mixed effect model of Experiment 2 (reaction times greater than mean + 3 × *SD* were excluded)

|  | **Degrees of freedom** | ***F* value** | ***p* value** |
| --- | --- | --- | --- |
| **repetition** | 1, 85 | 0.129 | 0.720 |
| **condition** | 2, 38443 | 2.045 | 0.129 |
| **ITI** | 1, 38464 | 361.924 | <0.001 |
| **interaction** | 2, 38430 | 3.006 | 0.049 |

The post hoc test shows the same significant difference in the estimated marginal means of linear trends between Condition P2 and S as well:

**Supplementary table 2** Post hoc tests of significant fixed effects. A, The estimated marginal means of linear trends from the interaction effects of condition and repetition number under the three conditions. B, Contrasts between conditions with *z* and *p* values

| **A,** | **EMM trend** | **SE** |
| --- | --- | --- |
| **S** | 0.00138 | 0.00216 |
| **P1** | 0.00013 | 0.00246 |
| **P2** | −0.00367 | 0.00246 |

| **B,** | ***z* value** | ***p* value** |
| --- | --- | --- |
| **S-P1** | 0.602 | 0.819 |
| **S-P2** | 2.442 | 0.039 |
| **P1-P2** | 1.594 | 0.248 |

The main difference between using mean + 2 × *SD* and mean + 3 × *SD* is that the latter reduces the statistical power of the models to 60%, so we decided to use the “stricter” 2 × *SD* criterion.
